# Assessing the palliative care needs of children with cancer and their families in tertiary care centres in India: A multicentre observational study

**DOI:** 10.1177/26323524261430380

**Published:** 2026-05-19

**Authors:** Vani Verma, Krithika S. Rao, Arunangshu Ghoshal, Vasudeva Bhat K, Harshitha D, Aishwarya Sanian, Aastha Mishra, Aditi Sakpal, Medha Bagalkote, Beda Sravani, Archana Iyengar, Sangeeta Mudaliar, Gayatri Palat, Veronique Dinand, Naveen Salins

**Affiliations:** 1 Department of Palliative Medicine and Supportive care, Kasturba Medical College, Manipal Academy of Higher Education, Manipal, India; 2 Department of Pediatric Oncology, Kasturba Medical College, Manipal Academy of Higher Education, Manipal,India; 3 Palliative and Supportive Care Unit, Bai Jerbai Wadia Hospital for Children, Parel, India; 4 Department of Pain and Palliative Medicine, MNJ Institute of Oncology and Regional Cancer Centre, Hyderabad, India

**Keywords:** paediatric oncology, palliative care, PaPaS, children with cancer, families, India

## Abstract

**Background:**

Children with cancer face a significant physical and psychosocial burden, highlighting the need for paediatric palliative care (PPC). Global estimates indicate that over 21 million children require PPC. However, in low- and middle-income countries such as India, the need is largely unknown, and access remains limited, necessitating the conduct of this study.

**Methods:**

A multicentre, prospective, cross-sectional study was conducted across three tertiary cancer centres in India to assess the palliative care needs of 150 children with cancer and their families using the Paediatric Palliative Screening Scale (PaPaS). The PaPaS tool evaluated five domains, and the total scores were used to stratify the need for PPC. The three participating centres differed in patient flow and the extent of palliative care integration, representing academic and public healthcare settings.

**Results:**

Based on PaPaS scores, 49.3% of children with cancer had moderate (secondary) palliative care needs, 36.7% required introduction to palliative care, 11.3% had minimal needs that paediatric oncologists could manage, and 2.7% required palliative care as the focus of treatment. Psychological distress was significantly higher among family caregivers (57%) than in patients themselves (33%). While 91% of families were open to palliative care discussions, clinicians perceived that only 4.7% (7/150) of children would likely benefit from referral to palliative care services. Domain-specific analysis revealed significant correlations between treatment burden, family distress, and overall palliative care needs.

**Conclusion:**

There is a significant unmet need for integrated PPC among children with cancer in India, exacerbated by a very small number of them accessing care due to non-referral by oncologists or the unavailability of PPC.

## Introduction

Paediatric palliative care (PPC) plays a vital role in supporting children with life-limiting or life-threatening conditions and their families by addressing their unique physical, emotional, and social needs.^1^ Unlike end-of-life only care, PPC can be introduced early and delivered alongside disease-directed treatment, particularly within paediatric oncology. Worldwide, approximately 21.6 million children live with conditions requiring palliative care, with 8.2 million needing specialised support.^1^ In India, the incidence of paediatric cancer is 38–124 per million under the age of 15 years, and less than 0.4% of those who require it have access to palliative care.^2^ Research indicates that end-of-life care for children with advanced cancer varies widely, underscoring the need for structured palliative care services. In paediatric oncology, the integration of palliative care is vital for comprehensive cancer treatment.^3,4^ Within paediatric oncology, palliative care complements routine oncology-led supportive care, which primarily focuses on managing treatment-related complications by addressing broader psychosocial and family-centred needs. This integration enhances children’s quality of life and supports their families during challenging times. Studies from various regions, including Indonesia, emphasise the importance of culturally sensitive approaches to palliative care that address specific local needs.^5^

One effective way to identify and address palliative care needs in children with cancer is through the Paediatric Palliative Screening Scale (PaPaS).^6^ This tool helps clinicians in assessing five key areas of need that align with the Spectrum of Needs framework, ensuring that children receive the appropriate level of support at the right time.^7,8^ By employing a structured scoring system, the PaPaS offers clear guidance on the introduction of palliative care. A score of 15 or higher indicates that a child may benefit from palliative care, facilitating timely interventions to enhance quality of life and alleviate the burden on families.^9,10^

Despite progress in recognising the importance of PPC, significant barriers persist, particularly in low- and middle-income countries.^2^ Limited resources, insufficiently trained healthcare professionals, and inadequate infrastructure pose ongoing challenges.^11,12^ However, the growing use of palliative care services among hospitalised children with cancer is a promising sign of progress towards more holistic and patient-focused care.^10,13–15^

India, home to one of the largest populations in the world, is facing a significant cancer burden. Nearly one million new cases are diagnosed each year, with over 80% detected at advanced stages. This highlights the urgent need for comprehensive palliative care services to support the increasing number of patients and families affected by advanced illness.^6,7,16^

To explore these challenges, we conducted a study using the PaPaS to assess the palliative care needs of 150 children with cancer and their families. This multicentric study is among the first to apply a structured tool to quantify PPC needs in India. By identifying gaps in care, we aim to guide the development of improved support systems and ensure timely and meaningful palliative care for vulnerable families.

## Methods

### Aim

This study aimed to assess the palliative care needs of children with cancer and their families using the PaPaS.^8^

### Study design

Between 1 January 2024 and 31 March 2024, the Departments of Palliative Medicine and Paediatric Oncology at Kasturba Medical College (KMC; Manipal), MNJ Institute of Oncology and Regional Cancer Centre (MNJIO and RCC), Hyderabad, and Bai Jerbai Wadia Hospital for Children (BJWHC), Mumbai, conducted a prospective cross-sectional multi-site survey study.

### Study setting

This multicentre study was carried out in three tertiary paediatric oncology centres across India, each representing a distinct healthcare context. At KMC, Manipal, a university teaching hospital in coastal Karnataka, paediatric oncology and palliative care services function side-by-side within an academic environment that emphasises multidisciplinary collaboration and research. The centre manages around 144 children each year, with about six monthly referrals to the palliative care team, reflecting a collaborative yet selective approach to palliative care integration. The MNJIO and RCC, Hyderabad, is a government-funded regional cancer centre that provides free or subsidised care to a large number of children from lower-income families. It has an established in-house palliative care department working closely with oncology units to extend support for symptom relief and counselling. Here, 350–450 children are seen annually, and all are reviewed daily by the PPC team, with a well-established PPC service structure within routine oncology practice. The BJWHC, Mumbai, is one of India’s oldest paediatric hospitals, serving children from both the city and nearby states. PPC services here operate through a consultative service approach in collaboration with oncology teams, focusing strongly on family-centred care and psychosocial support. The hospital treats about 200–300 children a year, with roughly one-fourth receiving palliative care support based on their individual needs.

*Inclusion and exclusion criteria:* Children aged 0–18 years with a confirmed cancer diagnosis (solid or haematologic) receiving active treatment or follow-up at KMC Manipal, MNJ Hyderabad, or Wadia Mumbai were included, along with their primary caregivers who provided consent. Children in remission for over a year, those with non-malignant conditions, or families unable to consent due to distress or language barriers were excluded. All eligible families consented to participate.

### Study population

A formal sample-size calculation was not performed because no prior Indian data were available to estimate the prevalence or effect size of palliative care needs using the PaPaS tool. Given the exploratory and feasibility nature of this multicentre study, a convenience sampling method was adopted, enrolling all eligible children with cancer and their caregivers attending the three centres during the 3-month study period (January–March 2024). This approach ensured consecutive recruitment within the study timeframe and provided preliminary insights to inform future, adequately powered studies.

### Data collection

A total of 150 children with cancer and their family caregivers (50 from each centre) were recruited. Data collection included basic demographic and clinical information, as well as structured interviews with caregivers. All eligible families consented to participate, likely reflecting the trust built with the treating teams and the non-invasive, interview-based nature of the study. Trained PPC specialists administered the PaPaS tool using a structured oral questionnaire.

The primary outcome measure was the PaPaS total score, representing overall palliative care need. Secondary outcomes included domain-wise PaPaS scores and item-level responses, including symptom burden, treatment burden, and caregiver-related concerns, which were examined descriptively and analytically to explore patterns associated with higher palliative care needs across centres.

### Definition of palliative care need categories

The PaPaS, developed by Bergstraesser et al.,^6,7^ helps clinicians identify when a child may benefit from palliative care. It looks at five key areas: The disease course, treatment burden, symptom intensity, family preferences, and expected life expectancy. Each area is scored, and the total score helps place children into one of four levels of need. To maintain consistency with international terminology, the categories were defined as follows: No or minimal needs (score <10): The child’s needs can be met within standard oncology care. Primary needs (10–14): Basic palliative care principles, such as early communication and symptom prevention, should be introduced alongside ongoing treatment.

Secondary needs (15–24): The child needs active support from a specialist palliative care team for complex symptom management and counselling. Palliative care as the focus (⩾25): The main goal shifts towards comfort and quality of life, with palliative care becoming the central approach. These definitions align with the Spectrum of Needs framework and reflect internationally accepted descriptions of palliative care intensity.^8^

### Statistical analysis

The collected data were first entered in Microsoft Excel and then analysed using Python (version 3.13.9) with the help of the Pandas, NumPy, and SciPy libraries. Continuous variables were described using the mean and standard deviation or the median and interquartile range, depending on the data distribution, while categorical variables were presented as numbers and percentages. The normality of data was checked using the Shapiro–Wilk test. To compare results between centres, we used one-way ANOVA for normally distributed data and the Kruskal–Wallis test for non-parametric data, followed by Bonferroni-adjusted post hoc tests where relevant. Relationships between clinical characteristics and PaPaS scores were examined using Spearman’s rank correlation coefficient (ρ) *p* < 0.05 was considered significant. A multiple linear regression model was also explored to identify predictors of higher palliative care needs, with checks to ensure that assumptions were met and multicollinearity was minimal (Variance Inflation Factor (VIF) < 5). Missing values from skip patterns within the PaPaS tool (e.g. item 4.2 answered only when 4.1 = “No”) were handled through pairwise deletion. All analyses were two-tailed, and results were considered statistically significant at *p* < 0.05. Multiple linear regression was performed as an exploratory, hypothesis-generating analysis to identify factors associated with higher PaPaS total scores; no causal inferences were intended.

Ethical approval was obtained from the institutional ethics committees of all three participating sites Kasturba Medical College and Kasturba Hospital Institutional Ethics Committee (IEC-198/2023), Institutional Ethics Committee MNJ Institute of Oncology and Regional Cancer Centre (ECR/227/Inst/AP/2013/RR-19), Institutional Ethics Committee Bai Jerbai Wadia Hospital for Children (IEC-BJWHC/61/2023). Confidentiality and data protection protocols were strictly adhered to throughout the study.

## Results

Our study involved 150 children diagnosed with cancer, recruited equally from three cancer centres in India: KMC, Manipal, a constituent unit of Manipal Academy of Higher Education (MAHE), Manipal. MNJIO and RCC, Hyderabad, and BJWHC, Mumbai (50 from each site; Table 1). The gender distribution was balanced (57.3% boys, 42.7% girls), and the largest age group was 4–12 years (60.7%; Table 2). The average age of the participating children was 8 years, while their caregivers, primarily parents, had a mean age of 34 years.

**Table 1.** Demographics of the WP1 participants.

Items	KMC, Manipal, *N* = 50	MNJ, Hyderabad, *N* = 50	WADIA, Mumbai, *N* = 50	Total, *N* = 150
Average age	10	7	7	8
Age caregiver	38	30	34	34
Gender	F-16, M-34	F-20, M-30	F-20, M-30	*F* = 64 (42.7%), *M* = 86 (57.3%)

KMC: Kasturba Medical College.

**Table 2.** Age distribution of paediatric population with cancer participating in the survey.

Age group	Number of patients
Infants (0–1 year)	8
Toddlers (>1–3 years)	18
Children (4–12 years)	91
Adolescents (13–18 years)	33

Among the cancer diagnoses, acute lymphoblastic leukaemia was identified as the most common, affecting 74 children (49%), followed by solid tumours in 56 children (37%; Table 3).

**Table 3.** Diagnosis of the survey participants.

Site	Acute lymphoblastic leukaemia	Solid tumours	Acute myeloblastic leukaemia	Langerhans cell histiocytosis	Acute megakaryocytic leukaemia	Mixed phenotypic leukaemia
Kasturba Medical College, Manipal	20 (40%)	24 (48%)	6 (12%)			
Wadia Children Hospital, Mumbai	32 (64%)	11 (22%)	4 (8%)	1 (2%)	1 (2%)	1 (2%)
MNJ Institute of Oncology, Hyderabad	22 (44%)	21 (42%)	7 (14%)			
Total	74 (49%)	56 (37%)	17 (11%)	1 (0.6%)	1 (0.6%)	1 (0.6%)

The caregiving landscape was predominantly composed of mothers, who served as the primary caregivers in 109 cases (72.7%), while fathers took on this role for 37 children (24.7%). In four instances, grandparents or other relatives fulfilled the caregiver role.

To assess the palliative care needs, we utilised the PaPaS, which classifies children into four groups based on their scores. Most children (74, 49.3%) were categorised as having moderate needs. Based on PaPaS categorisation, 36.7% had mild needs, 49.3% moderate, 11.3% low, and 2.7% high palliative care needs (Table 4).

**Table 4.** PaPaS stratification and scores.

PaPaS stratification	Number of participants
Does not need any input from paediatric palliative care (PaPaS score <10) # Extended survival years # No palliative care needs	17 (11.33%)
Introducing the concept of paediatric palliative care (PaPaS score 10–14) # Treatment might prolong life, not cure # Diminished survival # Palliative care needs ±	55 (36.66%)
Integrate with a paediatric palliative care team (PaPaS score 15–24) # Life expectancy <1 year # Treatment might prolong life, not cure # Palliative care needs +	74 (49.33%)
Palliative care is the focus of care (PaPaS score >25) # Life expectancy <6 months # High symptom burden # Rapid decline in performance status # High psychosocial distress	04 (2.66%)

PaPaS: Paediatric Palliative Screening Scale.

Regarding symptoms and psychological indicators, 60% of children experienced mild and manageable complaints, while only 33% exhibited psychological distress. However, distress was more frequently reported among caregivers, with 57% expressing emotional strain. A high rate of missing data (91%) was observed for Item 4.2, which is expected due to the skip logic of the PaPaS tool; clinicians completed this item only when families indicated “No” to Item 4.1 (Table 5).

**Table 5.** Domains of PaPaS score and scoring of the survey participants.

Domain and item numbers	Item	Characteristic	Scoring system	Count (%)
Domain 1	Trajectory of disease and impact on daily activities of the child			
1.1	Trajectory of disease and impact on daily activities of the child (in comparison with the child’s own baseline; with reference to the last 4 weeks)	Stable	0 □	60 (40%)
		Slowly deteriorating without impact on daily activities	1 □	67 (45%)
		Unstable with impact on and restriction of daily activities	2 □	20 (13%)
		Significant deterioration with severe restriction of daily activities	4 □	3 (2%)
1.2	Increase of hospital admissions (>50% within 3 months, compared to previous periods)	No	0 □	78 (52%)
		Yes	3 □	72 (48%)
Domain 2	Expected outcome of treatment directed at the disease and burden of treatment			
2.1	Treatment directed at the disease (does not mean treatment of disease related complications, such as pain, dyspnoea, or fatigue)	. . .is curative	0 □	37 (25%)
		. . .controls disease and prolongs life with good quality of life	1 □	93 (62%)
		. . .does not cure or control but has a positive effect on quality of life	2 □	18 (12%)
		. . .does not control and has no effect on quality of life	4 □	2 (1%)
2.2	Burden of treatment (Burden means side effects of treatment and additional burdens such as stay in hospital in the patient’s or family’s view)	No or minimal burden or no treatment is envisioned	0 □	17 (11%)
		Low level of burden	1 □	26 (17%)
		Medium level of burden	2 □	43 (29%)
		High level of burden	4 □	64 (43%)
Domain 3	Symptom and problem burden			
3.1	Symptom intensity or difficulty of symptom control (over the last 4 weeks)	Patients are asymptomatic	0 □	35 (23%)
		Symptom(s) are mild and easy to control	1 □	90 (60%)
		Any symptom is moderate and controllable	2 □	24 (16%)
		Any symptom is severe or difficult to control (unplanned hospitalisation or outpatient visits, symptom crises)	4 □	1 (1%)
3.2	Psychological distress of patient related to symptoms	Absent	0 □	30 (20%)
		Mild	1 □	49 (33%)
		Moderate	2 □	44 (29%)
		Significant	4 □	27 (18%)
3.3	Psychological distress of parents or family related to symptoms and suffering of the child	Absent	0 □	2 (1%)
		Mild	1 □	23 (15%)
		Moderate	2 □	40 (27%)
		Significant	4 □	85 (57%)
Domain 4	Preferences/needs of patient or parents Preferences of health professional			
4.1	Patient/parents wish to receive palliative care or formulate needs that are best met by palliative care	No	0 □ please answer 4.2	13 (9%)
		Yes	4 □ do not answer 4.2	137 (91%)
4.2	You/your team feel that this patient would benefit from palliative care	No	0 □	6 (4%)
		Yes	4 □	7 (missing 137; 5%)
Domain 5	Estimated life expectancy			
5.1	Estimated life expectancy	Several years Months to 1–2 years Weeks to months Days to weeks	0 □ please answer 5.2	125 (83%)
			1 □ please answer 5.2	22 (15%)
			3 □ do not answer 5.2	3 (2%)
			4 □ do not answer 5.2	0 (0%)
5.2	“Would you be surprised if this child were to suddenly die in 6 months time?”	Yes	0 □	129 (86%)
		No	2 □	18 (missing 3; 12%)

PaPaS: Paediatric Palliative Screening Scale.

Item-level findings from the PaPaS revealed that clinicians generally viewed the outlook for most children as favourable. Within the prognostic domain, 83% of children were considered likely to live for several years, and in 86% of cases, clinicians felt that an unexpected death within 6 months was unlikely. When the individual PaPaS items were correlated with total scores, the strongest associations emerged for treatment burden, symptom burden, and family psychological distress (*r* = 0.67–0.69), indicating that these factors showed the strongest associations with overall palliative care need. In contrast, family preference for palliative care showed only a weak relationship with total PaPaS scores (*r* = 0.14), suggesting that families’ expressed desire for palliative care support did not always mirror clinicians’ assessment of need (Table 6).

**Table 6.** Individual domain scores of PaPaS and their correlation with total scores.

Domain and item numbers	Item	Average score	Correlation with total score
Domain 1	Trajectory of disease and impact on daily activities of the child		
1.1	Trajectory of disease and impact on daily activities of the child (in comparison with the child’s own baseline; with reference to the last 4 weeks)	0.79	0.43
1.2	Increase in hospital admissions (>50% within 3 months, compared to previous periods)	1.44	0.56
Domain 2	Expected outcome of treatment directed at the disease and burden of treatment		
2.1	Treatment directed at the disease (does not mean treatment of disease-related complications, such as pain, dyspnoea, or fatigue)	0.91	0.35
2.2	Burden of treatment (Burden means side effects of treatment and additional burdens such as stay in hospital in the patient’s or family’s view)	2.45	0.69
Domain 3	Symptom and problem burden		
3.1	Symptom intensity or difficulty of symptom control (over the last 4 weeks)	0.95	0.49
3.2	Psychological distress of patient related to symptoms	1.63	0.67
3.3	Psychological distress of parents or family related to symptoms and suffering of the child	2.95	0.67
Domain 4	Preferences/needs of patient or parents Preferences of health professional		
4.1	Patient/parents wish to receive palliative care or formulate needs that are best met by palliative care	3.65	0.14
4.2	You/your team feel that this patient would benefit from palliative care	2.15	0.6
Domain 5	Estimated life expectancy		
5.1	Estimated life expectancy	0.21	0.37
5.2	“Would you be surprised if this child were to suddenly die in 6 months?”	0.24	0.29

PaPaS: Paediatric Palliative Screening Scale.

Upon examining site-specific differences, several statistically significant variations were observed (*p* < 0.05 across multiple variables). Differences were noted in the ages of children and caregivers, physician experience, perceptions of disease trajectory, frequency of hospitalisation, treatment burden, symptom intensity, psychological distress, and palliative care needs. These findings reflect site-level heterogeneity across the participating centres rather than comparative performance (Table 7).

**Table 7.** ANOVA results for site differences in PaPaS domains.

Metric	test type	Statistic	*p* Value
Trajectory of disease	ANOVA	6.778	<0.05
Increase in hospital admissions	ANOVA	3.973	<0.05
Treatment directed at disease	ANOVA	3.485	<0.05
Burden of treatment	ANOVA	78.163	<0.05
Symptom intensity	ANOVA	4.739	<0.05
Psychological distress (patient)	ANOVA	23.715	<0.05
Psychological distress (family)	ANOVA	105.241	<0.05
Palliative care request	ANOVA	12.905	<0.05
Estimated life expectancy	ANOVA	8.325	<0.05
Total	ANOVA	32.616	<0.05

PaPaS: Paediatric Palliative Screening Scale.

Comparisons were made across the three study sites Kasturba Medical College, Manipal; MNJ Institute of Oncology and Regional Cancer Centre, Hyderabad; and Bai Jerbai Wadia Hospital for Children, Mumbai.

**Figure 1.**
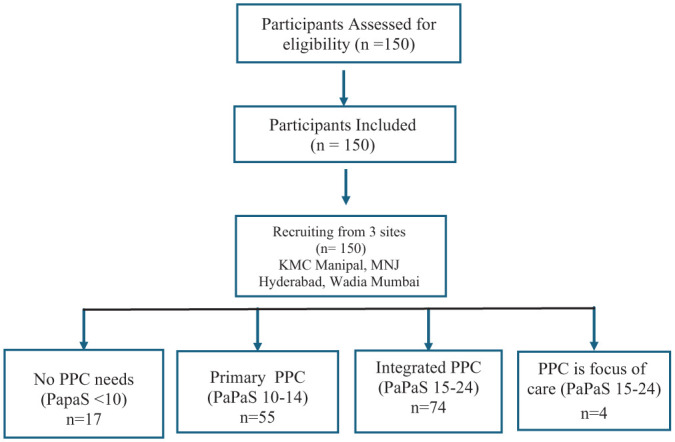
Flowchart of participant selection and stratification based on PaPaS scores. PaPaS: Paediatric Palliative Screening Scale.

Centre was included as a covariate to account for differences across sites, rather than to compare the performance of individual centres. In exploratory multivariable analysis, higher PaPaS scores were associated with greater symptom burden (Item 3.2) and selected caregiver-related variables. Given the high proportion of missing data for Item 4.2 due to skip logic, findings related to caregiver preference should be interpreted cautiously. The recruitment process is summarised in Figure 1.

## Discussion

This study offers insights into the palliative care needs of children with cancer and their families in India, highlighting the varying levels of support required, the burden of treatment and indications of psychological distress among caregivers. This study assessed palliative care needs using a structured screening tool and was not designed to evaluate or compare the effectiveness, timing, or impact of specific models of PPC delivery. Participation in PaPaS screening represented consent to needs assessment and should not be interpreted as acceptance of PPC services or referral. We categorised children based on the severity of their palliative care needs to help prioritise support, most showing secondary/specialist palliative care needs and a few requiring palliative care as the focus of treatment. In contrast, Kaye et al. advocate for early palliative care from the time of diagnosis for all high-risk cases, focusing on relationship-building and continuous support. Unlike earlier reports that describe the general value of early palliative care, our study uniquely quantifies how levels of need vary across settings and highlights a clinician-family perception gap regarding who is most likely to benefit from PPC. Similar unmet palliative care needs have been documented in high-income settings, including the Italian experience described by Chelazzi et al.,^17^ where persistent symptom burden, delayed referrals, and structural barriers to early integration of PPC were noted despite well-established health systems. While such work primarily offers narrative and system-level perspectives, our study complements this literature by providing patient-level, needs-based evidence derived from structured screening within routine oncology care. In doing so, our findings contextualise global evidence within a low-middle-income country setting and illustrate how systematic screening may facilitate earlier identification of palliative care needs beyond traditional referral-driven approaches.^17^ This gap underscores the importance of systematic screening and early dialogue within oncology practice. While our approach identifies who needs care most urgently, Kaye’s model highlights the potential value of early palliative care involvement across the illness trajectory.^18^ This study was conducted at three tertiary centres representing academic, public healthcare settings; the findings offer a meaningful glimpse into the broader landscape of PPC in India. Items within the PaPaS included skip patterns that were analysed; accordingly, for instance, clinicians completed Item 4.2 (“Clinician perceives benefit from palliative care”) only when families responded “No” to Item 4.1 (“Family wishes for palliative care”). This design led to some missing responses but ensured contextual accuracy in paired questions. Our findings align with those of Muckaden et al.,^19^ who highlighted the significant unmet need for PPC in Maharashtra and showed that integrating PPC within paediatric oncology services can greatly improve access, communication, and family support.^19^

Our observations also resonate with the findings of Bisen et al.,^20^ who described the clinical and socio-demographic profile of children receiving PPC in a tertiary Indian hospital and noted that most referrals occurred late in the disease course. Their results underscore the persistent gap between those who need palliative care and those who receive it.^20^ Observed centre-level differences are likely to reflect variations in institutional context, patient mix, and care practices rather than differences in centre performance, underscoring the contextual nature of palliative care needs across settings.

Our study identified a communication gap between families and clinicians, with many families open to palliative care discussions but few teams initiating them. Consistent with Salins et al. and Snaman et al., strengthening communication through structured clinician training can improve trust, shared decision-making, and overall quality of care within paediatric oncology.^18,21,22^ In the context of our needs-based screening approach, communication should not occur only when needs become urgent but rather be integrated early and revisited across the disease trajectory, ensuring that information, expectations, and decisions evolve with the child’s condition.^21^

Our study shows that childhood cancer doesn’t just affect the child; it deeply impacts the whole family, especially the caregivers. Caregiver-related screening domains indicated emotional strain, particularly in the context of pain management and uncertainty, consistent with findings from India and other Low-and middle-income countries (LMICs) showing that families face unmet emotional, social, and spiritual needs when PPC services are limited.^22–26^ All of these point to a clear message caregivers need more support. Mental health counselling, support groups, and programs that help them build emotional strength can make a big difference not just for them, but for the children, too.^27,28^ Frequent hospital admissions were identified as a key factor influencing palliative care needs. Among all 150 children assessed, nearly 48% of children had increased hospital admissions in the past 3 months.

Our needs-based screening approach complements this approach by identifying those who require early and intensified palliative care support, ensuring timely referral without delaying universal integration of PPC principles. When palliative care is introduced early, as shown in studies by Plessis et al. and Salins et al, it helps manage these symptoms better from the beginning.^29,30^ It also gives families emotional support, improves communication with doctors, and helps guide tough decisions. Rather than waiting for a crisis, early palliative care walks alongside the family throughout the illness, reducing stress and improving the overall experience of care.^30,31^ Delayed referrals to palliative care can lead to unmanaged symptoms, increased caregiver distress, and poor quality of life for children.^21^ Several studies have identified predictors of late palliative care referrals, including lack of awareness, provider hesitancy, and the misconception that palliative care is only for end-of-life situations.^32,33^ However, when introduced early, palliative care provides holistic support, improves symptom control, and enhances family decision-making.^22,29,34^

These findings suggest several practical implications for paediatric oncology care. While palliative care needs in children with cancer are well documented in high-income settings, comparable evidence from India and other low-middle-income countries remains limited. This multicentre study provides a needs-based assessment across diverse Indian oncology settings, demonstrating a high burden of unmet palliative care needs and caregiver distress despite variation in service availability. Our findings align with earlier Indian evidence showing that PPC is often introduced late and largely at advanced stages of illness. However, unlike prior referral-based studies such as that by Ghoshal et al., which describe palliative care involvement following clinical deterioration, this study identifies unmet needs earlier through structured screening. By contextualising global evidence within an LMIC setting, these findings highlight the potential role of routine needs-based screening in enabling earlier symptom management and more timely integration of PPC, rather than reliance on late referral-driven models.^21^ First, systematic early screening for palliative care needs should be incorporated into routine care to ensure that children and families receive appropriate support at the right time.^30,35^ Second, healthcare providers should be trained in palliative care communication to ensure that conversations about PPC are introduced sensitively and effectively.^18,32^ Third, expanding home-based palliative care services could help reduce hospital admissions and provide children with a more comfortable, family-centred care experience.^12,29^ These findings highlight the importance of recognising caregiver-related psychological concerns and may inform the need for structured support approaches tailored to family challenges.^24,26,36^

### Limitations

Although this study provides critical insights into palliative care needs in paediatric oncology, certain limitations should be considered. The cross-sectional design restricts the ability to assess how palliative care needs evolve, highlighting the necessity for longitudinal studies.^18,22^ In addition, caregiver-reported outcomes may introduce response bias, as perceptions of symptom burden can be subjective.^37^ Lastly, the study would have benefited from qualitative data to provide deeper insights into family experiences and decision-making processes related to palliative care.^29^ No power analysis was conducted this limits generalisability and precision of estimates. High missingness in Item 4.2 due to skip logic limits interpretation.

PaPaS has not been formally validated in the Indian context, and no linguistic translation or cultural adaptation was undertaken as part of this study. This represents a limitation. However, as PaPaS was used for needs screening rather than outcome measurement and was administered by trained clinicians in routine clinical settings, this limitation is unlikely to undermine its utility for identifying palliative care needs in the present context.

Future research should prioritise longitudinal studies to better characterise how palliative care needs evolve over time and to clarify the timing of palliative care involvement across the illness trajectory.^12,32^ In addition, interventional studies could examine whether earlier referral to PPC is associated with differences in symptom burden, caregiver-related distress, and quality of life outcomes.^22,33^ Further research is also warranted to explore approaches for strengthening communication and shared decision-making between families and healthcare teams.^18,30^ Studies evaluating different modes of delivering palliative care support, including home-based services, may provide insights into patterns of hospital utilisation and patient–family experiences.^21,24,25,29,35,38^ Finally, as this study was conducted across three tertiary care centres, variations in institutional context and care practices may have influenced the observed palliative care needs; therefore, the findings should be interpreted cautiously when extrapolating to other paediatric oncology settings in India, particularly those with limited resources or community-based care structures.

## Conclusion

This multicentre study sheds light on the varied palliative care needs faced by children with cancer and their families across three major oncology centres in India. Almost half of the children were identified as having higher palliative care needs, and caregiver-related screening domains indicated emotional strain. A clear gap emerged between family preferences and clinician perceptions, highlighting communication challenges that may influence care decisions. Frequent hospital visits and treatment burden were closely associated with higher palliative care needs. Together, these findings underscore the importance of early, systematic identification of palliative care needs and highlight implications for holistic, family-centred support across the illness trajectory.

## Supplemental Material

sj-docx-1-pcr-10.1177_26323524261430380 – Supplemental material for Assessing the palliative care needs of children with cancer and their families in tertiary care centres in India: A multicentre observational study Supplemental material, sj-docx-1-pcr-10.1177_26323524261430380 for Assessing the palliative care needs of children with cancer and their families in tertiary care centres in India: A multicentre observational study by Vani Verma, Krithika S. Rao, Arunangshu Ghoshal, Vasudeva Bhat K, Harshitha D, Aishwarya Sanian, Aastha Mishra, Aditi Sakpal, Medha Bagalkote, Beda Sravani, Archana Iyengar, Sangeeta Mudaliar, Gayatri Palat, Veronique Dinand and Naveen Salins in Palliative Care and Social Practice
